# The Origins and Roles of Methylthiolated Cytokinins: Evidence From Among Life Kingdoms

**DOI:** 10.3389/fcell.2020.605672

**Published:** 2020-11-09

**Authors:** Maya Gibb, Anna B. Kisiala, Erin N. Morrison, R. J. Neil Emery

**Affiliations:** Department of Biology, Trent University, Peterborough, ON, Canada

**Keywords:** 2MeSZ, 2MeSiP, methylthiolated cytokinins, methylthiotransferase, tRNA degradation pathway

## Abstract

Cytokinins (CKs) are a group of adenine-derived, small signaling molecules of crucial importance for growth and multiple developmental processes in plants. Biological roles of classical CKs: isopentenyladenine (iP), *trans* and *cis* isomers of zeatin (*t*Z, *c*Z), and dihydrozeatin, have been studied extensively and their functions are well defined in many aspects of plant physiology. In parallel, extensive knowledge exists for genes involved in tRNA modifications that lead to the production of tRNA-bound methylthiolated CKs, especially in bacterial and mammalian systems. However, not much is known about the origins, fates, and possible functions of the unbound methylthiolated CKs (2MeS-CKs) in biological systems. 2MeS-CKs are the free base or riboside derivatives of iP or Z-type CKs, modified by the addition of a thiol group (–SH) at position 2 of the adenine ring that is subsequently methylated. Based on the evidence to date, these distinctive CK conjugates are derived exclusively via the tRNA degradation pathway. This review summarizes the knowledge on the probable steps involved in the biosynthesis of unbound 2MeS-CKs across diverse kingdoms of life. Furthermore, it provides examples of CK profiles of organisms from which the presence of 2MeS-CKs have been detected and confirms a close association and balance between the production of classical CKs and 2MeS-CKs. Finally, it discusses available reports regarding the possible physiological functions of 2MeS-CKs in different biological systems.

## Introduction

Cytokinins (CKs) are a group of adenine-derived, small signaling molecules, that comprise a class of phytohormones that are of crucial importance for multiple growth and developmental processes in plants (Spíchal, 2012; Kieber and Schaller, 2018). Plant CKs play a significant role in regulating cell proliferation and differentiation, control of shoot/root balance, transduction of nutritional signals (source/sink distribution), delaying senescence, and increasing crop productivity (Sakakibara, 2006). Cytokinins exist in two main structural forms, depending on the chemistry of the side chain attached at N^6^ position to the adenine ring – isoprenoid or aromatic. Regarding the structure of their side chain, classical, isoprenoid CKs include isopentenyladenine (iP), zeatin (*trans* and *cis* isomers; *t*Z, *c*Z), dihydrozeatin (DZ), and their various derivatives and conjugates (Kisiala et al., 2019) that often strongly differ in their biological activity (Spíchal, 2012).

In plants, isoprenoid CKs can be synthesized via two metabolic pathways, the *de novo* pathway, and the tRNA degradation pathway. In *de novo* CK biosynthesis, specific isopentenyltransferase (IPT) enzymes (adenylate IPTs) add the isopentenyl sidechains (originating mainly from the methylerythritol phosphate pathway; MEP) to adenosine tri- di- or monophosphates (ATP, ADP, AMP). The *de novo* pathway, that is often a main source of plant CKs, is localized mainly in cell plastids and results predominantly in the production of iP- and *t*Z-type CKs that typically demonstrate high biological activity (Sakakibara, 2006).

The other known CK production pathway involves a modification of the adenine base at position 37 of tRNA molecules with the isopentenyl sidechain obtained primarily via the mevalonate pathway (MVA). Production of CKs through tRNA degradation is localized mainly in the cytosol. Generally, tRNA modifications contribute to an increased adaptation to environmental conditions through the control of translational efficiency and fidelity, in addition to reading frame maintenance (Dabravolski, 2020; Liaqat et al., 2020). Following the degradation of the modified tRNA molecules, *c*Z-type CKs are produced. Although the tRNA degradation pathway is thought to play only a minor role in overall CK production, it contributes significantly to the levels of *c*Z-CKs in certain plant species (Emery et al., 1998; Frébort et al., 2011; Gajdošová et al., 2011).

The presence of CK metabolites, or the genetic mechanisms required for CK production, have been reported in all kingdoms of life (Björk, 1995; Mayaka et al., 2019). Cytokinin biosynthesis involves conserved mechanisms even among evolutionary distant organisms, and the formation of CKs in bacteria, fungi, plants, or mammals all involve specific gene homologs and their corresponding enzymes, including IPT, adenosine kinase (AK), LONELY GUY (LOG) and a CK-degradation enzyme, cytokinin oxidase/dehydrogenase (CKX; Sakakibara, 2006; Chanclud et al., 2016; Trdá et al., 2017; Daudu et al., 2019). Genes encoding enzymes responsible for subsequent steps of CK metabolism exist in a variety of different organisms, although not all genes have been fully characterized or discovered.

**FIGURE 1:**
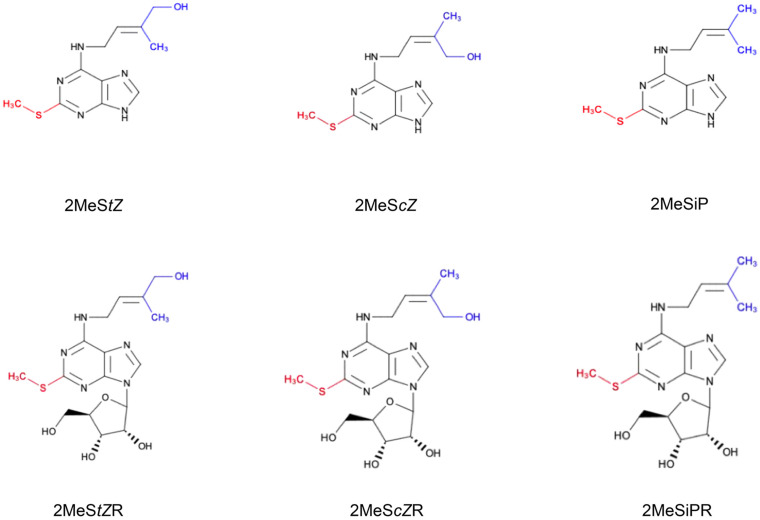
Chemical structures of methylthiolated cytokinins (2MeS-CKs). The attachment of the –S–CH_3_ group (red) at C_2_ position of the adenine ring of tRNA-bound CKs is regulated by methylthiotransferase-like enzymes. 2MeS-CK forms differ based on the modifications to their isoprenoid side chain (blue) and include: 2-methylthio-*trans*Zeatin (2MeS*t*Z), 2-methylthio-*cis*Zeatin (2MeS*c*Z), and 2-methylthio-isopentenyladenine (2MeSiP), and their riboside derivatives: 2MeS*t*ZR, 2MeS*c*ZR and 2MeSiPR, respectively.

Since the end of the 20th century, significant discoveries in plants have been made; from not even knowing if plants synthesized their own CKs (Holland, 1997), to having nearly comprehensively defined CK pathways, leading to identification of the active CKs and their corresponding conjugates (e.g., *cis* and *trans*-zeatin, iP, DZ and nucleotide, riboside, glucoside conjugates) (Kieber and Schaller, 2018). Unknown CK pathways are now rare and include those that may produce aromatic side chain CKs (Frébort et al., 2011) and the seldomly observed conjugates such as lupinic acid, a zeatin metabolite isolated from *Lupinus angustifolius* seedlings (Guern and Peaud-Lenoel, 2012), mono- and dimethylated isopentenyladenine CKs found in virulent *Rhodococcus fascians* strains (Jameson, 2019) or discadenine, iP derivative unique for slime mold *Dictyostelium discoideum* (Aoki et al., 2020). Another curious case is that of the hydrophobic, methylthiolated CKs (2MeS-CKs) (Figure 1). 2MeS-CKs are iP- or Z-type CKs modified by addition of a thiol group (–SH) at the position 2 of the adenine ring and its subsequent methylation (Tarkowski et al., 2010). Based on the evidence to date, these unique CK conjugates are derived exclusively via the tRNA degradation pathway (Koenig et al., 2002; Morrison et al., 2015a, 2017). 2MeS-CKs are commonly observed, yet they are poorly understood in terms of their origins, biological activity and functions. Due to their low quantities in plant tissues, the development of analytical methods with adequate sensitivity to detect these compounds is essential in order to elucidate their biological function (Tarkowski et al., 2010). A survey of CK review literature has yet to offer a place for 2MeS-CK production in the overall CK pathway schemes (Spíchal, 2012; Morrison et al., 2015a, 2017).

This review will summarize the knowledge on the probable steps involved in biosynthesis of the unbound 2MeS-CKs. Furthermore, it will provide examples of CK profiles for organisms from which 2MeS-CKs were previously detected and confirm a close association and balance between production of classical CKs and 2MeS-CKs. Finally, it will discuss available reports regarding any possible physiological functions of 2MeS-CKs in different biological systems.

## The Steps of 2MeS-CK Production via the tRNA Degradation Pathway

### The Biosynthesis of the Initial CKs via tRNA Modification: tRNA Dimethylallyltransferase (EC 2.5.1.75): MiaA/MOD5/AtIPT2 and 9/TRIT1

**FIGURE 2:**
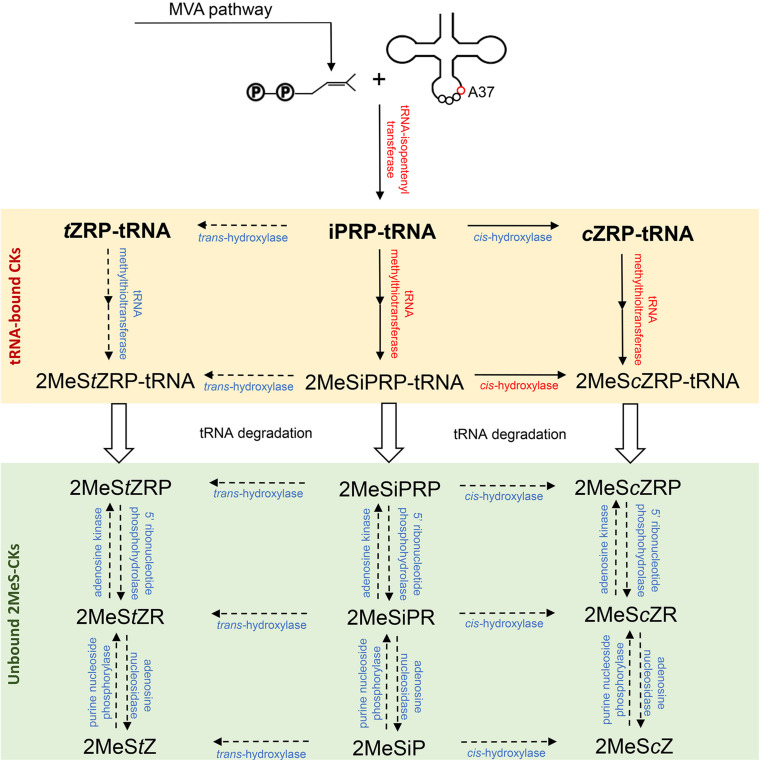
Biosynthesis pathway of the methylthiolated cytokinins (2MeS-CKs) proposed based on the microbial, plant and animal 2MeS-CK metabolite profiles obtained at the Water Quality Centre, Trent University and from other laboratories. Three groups of enzymes (red font) involved in the production of 2MeS-CKs have been previously identified across kingdoms of life including: tRNA-isopentenyltransferases (e.g., miaA, MOD5, IPT2 and 9, TRIT1), tRNA-methylthiotransferases (e.g., miaB + C, CDK5RAP1), and cis-hydroxylases (e.g., miaE) (Dabravolski, 2020). The inferred enzymes involved in further modification of tRNA-bound and free 2MeS-CKs (blue font, dashed arrows) were predicted based on the information available from other CK producing pathways (Sakakibara, 2006; Spíchal, 2012; Morrison et al., 2017; Aoki et al., 2019a). Mevalonate (MVA) pathway is the main source of the isoprenoid substrate for tRNA-isopentenyltranserases; however, a small pool of the prenyl chain molecules can originate from the methylerythritol phosphate (MEP) pathway. If tRNA degradation occurs prior to methylthiolation of the prenylated adenine nucleobase (A37), tRNA-bound iPRP, tZRP, and cZRP (bold font) are released and act as the precursors of classical CKs (iP, *t*Z and *c*Z).

The first step toward 2MeS-CK production involves the initial formation of a CK in the tRNA degradation pathway. It involves the addition of an isopentenyl group to an adenine at position 37 of tRNA molecules, which is known to read codons beginning with uridine (Figure 2). This isopentenyl addition occurs via the tRNA-isopentenyltransferase enzyme (tRNA-IPT) and it creates a tRNA-bound N^6^-isopentenyladenosine phosphate (iPRP). It also represents the rate limiting step of CK biosynthesis. To date, tRNA-IPT homologs have been identified in bacteria, fungi, plants, insects, and mammals (Pertry et al., 2009; Dabravolski, 2020).

In bacteria, the enzyme responsible for tRNA isopentenylation is MiaA (Buck and Ames, 1984; Gray et al., 1996; Koenig et al., 2002). A deletion of the *miaA* gene in *Salmonella typhimurium*, a pathogenic, Gram-negative bacterium, caused extensive pleiotropic effects, including a temperature-sensitive growth phenotype (Blum, 1988). *miaA* mutant strain of *Bradorhizobium* spp. displayed a significant reduction in CK production but was also characterized by more intense growth in CK-free media (Podlešáková et al., 2013). The isopentenyl side chain of the tRNA-bound iP can be further modified via *cis*-hydroxylase, forming a tRNA-bound *cis* isomer of Zeatin (tRNA-*c*Z) and, upon tRNA degradation, iP- or *c*Z-type CKs are released (Cherayil and Lipsett, 1977; Morris et al., 1981; McGaw and Burch, 1995). tRNA-derived *c*Z-CKs are often thought to have significantly reduced biological activity, compared to the *de novo* synthesized, highly active *t*Z-type CKs (Sakakibara, 2006; Wang et al., 2020); however, recent studies suggested *c*Z is strongly involved in plant stress alleviation (Gajdošová et al., 2011; Schäfer et al., 2015; Silva-Navas et al., 2019). *trans-*zeatin secretion in plant symbiotic *Methylobacterium* was previously linked to a tRNA source since the presence of Z *trans*-isomers was detected even in the absence of the genetic machinery for *de novo* pathway synthesis. The tRNA hydrolysate of the *Methylobacterium extorquens miaA* mutant also lacked the precursor riboside forms, *trans*-zeatin riboside (*t*ZR) and isopentenyladenosine (iPR) (Koenig et al., 2002). The presence of significant levels of *t*Z and its derivatives have since been reported from a wide selection of plant-associated *Methylobacterium* strains (Jorge et al., 2019), additionally supporting the claim that, unlike plants, some bacteria can derive *t*Z isomers via a tRNA source. The possibility of obtaining *t*Z-type CKs via the tRNA-IPT activity was also proposed for a phytopathogenic fungi-plant interaction system (Morrison et al., 2017) while production of *t*Z-type CK via *de novo* biosynthesis pathway has been previously identified in a fungal rye pathogen, ergot (*Claviceps purpurea*) (Hinsch et al., 2015).

The dominance of *c*Z- and iP-type CKs in the hormone profiles of saprophytic and biotrophic fungal species suggests that the tRNA degradation is the predominant CK pathway in that kingdom (Morrison et al., 2015b; Moffatt et al., 2018). A fungal tRNA-isopentenyltransferase gene homolog (*MOD5*) was first identified in yeast (*Saccharomyces cerevisiae*) (Laten et al., 1978; Dihanich et al., 1987). Evidence to support its function in CK production was demonstrated via a Mod5-1 mutation in *S. cerevisiae* that caused a significant reduction in biosynthesis of iPR from cytoplasmic and mitochondrial tRNA (Laten et al., 1978). Mod5, a yeast tRNA-isopentenyltransferase, is also involved in protein synthesis and sporulation (Laten et al., 1978) and Mod5 mutants displayed reduced resistance to antifungal agents (Suzuki et al., 2012). More recently, a survey confirmed production of zeatin-type CKs in a wide range of yeast species (Streletskii et al., 2019); however, the authors did not resolve their data between the *trans*- and *cis*-isomers.

tRNA-IPT genes responsible for CK biosynthesis have been identified in pathogenic fungi and fungal CK production is a crucial factor in strain virulence (Chanclud et al., 2016; Morrison et al., 2017; Trdá et al., 2017). Cytokinin Synthesis 1 (*CKS1*), a gene encoding a tRNA-IPT was identified in the rice pathogen, *Magnaporthe oryzae* (Chanclud et al., 2016). *cks1* mutants showed normal *in vitro* growth; however, the deletion resulted in impaired CK production preventing the mutants from maintaining nutrient levels at the site of infection. This in turn, led to the induction of early and strong plant defenses suggesting that fungal CKs contribute to metabolite mobilization and to rice defense inhibition (Chanclud et al., 2016). In *Ustilago maydis*, a biotrophic fungal pathogen that causes corn smut disease, the tRNA-IPT homolog was designated as *UMAG_10043* (Morrison et al., 2017). Deletion of *UMAG_10043* resulted in a loss of CK production and significantly reduced virulence of the fungus during infection of maize seedlings and cobs but it also affected fungus filamentous growth and its ability to modify CKs taken up from the media (Morrison et al., 2017). The analysis of *LmIPT* (XP_003842057) silenced lines of *Leptosphaeria maculans*, a hemibiotrophic fungal pathogen of oilseed rape, demonstrated a significant reduction in *c*Z levels (Trdá et al., 2017). However, no other phenotypic effects were observed in the mutants, and total CK levels were not altered, suggesting that another, alternative CK biosynthesis pathway might be active in *L. maculans* (Trdá et al., 2017).

Another microbial CK producer, slime mold (*Dictyostelium discoideum*) is a member of the *Amoebozoa* phylum and possesses three IPT genes, two of which are putative tRNA-IPT genes (*iptB* and *iptC*) (Anjard and Loomis, 2008; Nishii et al., 2018). These tRNA-IPT gene candidates are thought to be distantly related to plant IPTs and they were acquired in *Dictyostelium* by horizontal gene transfer (Eichinger et al., 2005; Nishii et al., 2018). Although the biological role of the two putative tRNA-IPTs in *D. discoideum* have not been confirmed, the available CK profiles suggest they might be involved in the production of *c*Z-type and 2Me-S-type CKs by slime mold (Aoki et al., 2019a).

In plants, CKs contribute an important role in inducing plant cell division, differentiation and regulation of various aspects of plant development and interaction with the biotic and abiotic environment (Sakakibara, 2006; Kieber and Schaller, 2018). tRNA-IPT homologs were identified in *Physcomitrella patens*, a moss species that belongs to the early divergent clade of land plants utilizing CKs for growth control (Lindner et al., 2014). In *Arabidopsis thaliana*, 9 isopentenyltransferase genes have been identified (*AtIPT1-9*), although only two; *AtIPT2* (AT2G27760) and *AtIPT9* (AT5G20040) are known to be responsible for modifying tRNA and production of *c*Z type CKs (Miyawaki et al., 2006). Although *c*Z activity is greatly limited in comparison with *t*Z or iP type CKs, the evidence supports the importance of plant tRNA-IPTs. For example, the *atipt2 9* double mutants demonstrate reduced *c*Z CK levels, but their iP and *t*Z-type CKs are not affected, and yet they often have a chlorotic phenotype (Miyawaki et al., 2006).

The available studies of CKs in the kingdom of Animalia indicate the existence of an active tRNA pathway in insects and mammals. Cytokinin metabolites have been found in insects (e.g., Straka et al., 2010; Andreas et al., 2020); although, it has been postulated that CK presence in insects might be a result of the activity of bacterial symbionts (Giron et al., 2013). In silkworm (*Bombyx mori*), a candidate tRNA-IPT gene with three alternative splicing isoforms (*BmIPT1-BmITP3*) was recently identified (Chen et al., 2019). A recombinant vector containing *BmIPT1* could restore isopentenylation of tRNA in the IPT-deficient yeast strain MT-8 suggesting BmIPT1 is a functional tRNA-IPT enzyme in *B. mori*. Although no CK data are available for silkworm to date, the importance of BmIPT1 was demonstrated following the i^6^A (iPR) modification at position A37 in tRNA, which resulted in severe abnormalities in silk spinning and metamorphosis (Chen et al., 2019).

Cytokinin presence and metabolism has been reported in canine tissues (Seegobin et al., 2018) and human cell cultures (Aoki et al., 2019b), and the CK profiles indicate that tRNA degradation is the only source of unbound mammalian CKs. tRNA-isopentenyltransferase 1 (*TRIT1*), a homolog of the prokaryotic *miaA*, facilitates production of tRNA-bound iP-type CKs in human cytosol and mitochondria (Lamichhane et al., 2013; Smaldino et al., 2015). *TRIT1* was previously suggested to play a role as a tumor suppressor (Spinola et al., 2005), in selenoprotein regulation (Fradejas et al., 2013) in gene-mediated transcriptional silencing (Smaldino et al., 2015) and amyloid fiber folding (Waller et al., 2017). The product of the *TRIT1* gene, iPR, has long been the lone CK type detected as tRNA-bound or as a free mononucleotide in mammals (Persson et al., 1994; Golovko et al., 2000). Since then, more recent studies on CK profiling by High Performance Liquid Chromatography Tandem Mass Spectrometry (HPLC-MS/MS) revealed a presence of seven types of CKs in a range of canine tissues (Seegobin et al., 2018). The presence of unbound CK derivatives in HeLa cell cultures were detected, strengthening the evidence of TRIT1 activity in CK biosynthesis in mammalian cells (Aoki et al., 2019b).

### The First Step of Methylthiolation of tRNA-Bound CKs: tRNA-2-Methylthio-N(6)-Dimethylallyladenosine Synthase (EC 2.8.4.3): MiaB + MiaC/AT4G36390/CDK5RAP1

Following isopentenylation, methylthiolation of the adenine ring at the C_2_ position is the next step of the tRNA degradation pathway that leads to the production of 2MeS-CKs (Figure 2). In bacteria, the gene responsible for methylthiolation is *miaB* which modifies tRNA-bound i^6^A (iPR) or io^6^A (*cis*Zeatin riboside; *c*ZR) to ms^2^i^6^A (2-methylthio-isopentenyladenine riboside; 2MeSiPR) and ms^2^io^6^A (2-methylthio-*cis*Zeatin riboside; 2MeS*c*ZR), respectively (Esberg et al., 1999; more details on hydroxylation of the isoprenoid chain of tRNA-bound CKs will be provided in the section “Hydroxylation of the Isopentenyl Chain of tRNA-Bound 2MeSiP-CKs: tRNA-ms2io6A37-Hydroxylase: MiaE/CYP450”). Methylthiolation requires iron (Fe), cysteine (Cys), S-adenosylmethionine (SAM) and is thought to occur in two steps, initially as thiolation of i^6^A37 (tRNA-bound iPR) to s^2^i^6^A37, and a subsequent methyl transfer gives ms^2^i^6^A37 (tRNA-bound 2MeSiPR) (Esberg et al., 1999). It remains unknown whether each reaction is catalyzed by a single enzyme (MiaB + MiaC) or both steps are facilitated by the same enzyme, MiaB (Pierrel et al., 2003). However, *Escherichia coli* mutant strains that lacked a functional *miaB* gene were shown to contain only the product of the first step in the tRNA pathway, i^6^A37; thus it is suggested that the MiaB protein is involved in formation of the C-S bond that can be further methylated via *MiaC* activity (Esberg et al., 1999). A conserved canonical Cys triad is found both in the N-terminal half of *miaB* genes and in enzymes such as biotin and lipoate synthases, which are involved in catalyzing C–H to C-S bond conversion reactions. The motif provides Cys ligands for a [4Fe-4S]^+2/+1^ cluster. The Cys triad and iron cluster are essential for thiolation activity which indicates that MiaB, biotin synthase, and lipoate synthase all utilize similar radical mechanisms to activate sulfur (S) and insert it into the respective substrates (Pierrel et al., 2002). Additionally, MiaB enzymes contain a sequence of 60–80 residues in the C-terminal region that is similar to the ß-barrel RNA-binding domains (Anantharaman et al., 2001). This domain, known as TRAM, was identified as the site likely involved in binding the tRNA substrate (Pierrel et al., 2003). The level of synthesis of the 2-methylthiol group of ms^2^io^6^A (2MeS*c*ZR) is sensitive to the presence of S or Fe and may function as a signal device for the availability of these elements (Buck and Griffiths, 1982; Buck and Ames, 1984).

The role of the *miaC* gene has been postulated to exist but never identified (Pierrel et al., 2004). MiaC is thought to function during the methylthiolation reaction as a methyltransferase following the S transfer performed by MiaB. Examples of methyltransferase genes in plant pathogenic bacteria can be found in *Streptomyces turgidiscabies* and *R. fascians* which both carry a *fas* operon (Joshi and Loria, 2007; Pertry et al., 2009). The *fas* operon contains two open reading frames (ORFs) that code for methyltransferases (*mtr-1* and *mtr-2*) which may be involved in the methylthiolation of bacterial CKs (Frébort et al., 2011).

Unlike bacteria, there are no *miaB* homologs yet identified from fungi. Correspondingly, 2MeS-CKs have not been found in axenic fungal cultures (Table 1; Hinsch et al., 2015; Chanclud et al., 2016; Morrison et al., 2017; Vedenicheva et al., 2018). Previously, 2MeS-CKs were detected from temperate forest fungi collected *in situ* (Morrison et al., 2015b); however, these samples were not grown aseptically and would have had an associated microbiome, including bacteria capable of producing 2MeS-CKs.

**TABLE 1:** Profiles of methylthiolated cytokinins (2MeS-CKs) among representatives of different life kingdoms.

**Kingdom**	**Species**	**2MeS-CKs detected**	**2MeS-CK level**	**Total CK level**	**References**
Bacteria	*Erwinia amylovora* CFBP1430	2MeSZ, 2MeSiP, 2MeSiPR	1–5 pmol/mL	10–20 pmol/mL	Daudu et al., 2019
	*Methylobacterium jeotgali* LMG23639(T)	2MeSZ, 2MeSZR	5–10 pmol/mL	10–20 pmol/mL	Jorge et al., 2019
Protists	Slime mold (*Dictyostelium discoideum*) (aggregation)	2MeSiP	1–5 pmol/10^6^cells	1–5 pmol/10^6^cells	Aoki et al., 2019a
	*Euglena gracilis* (cell pellet)	MeSiP	1-5 pmol/gFW	20–50 pmol/gFW	Noble et al., 2014
Fungi	*Magnaporthe oryzae* (mycelia)	n.d.	n.d.	150–200 pmol/gFW	Chanclud et al., 2016
	*Ustilago maydis* (mycelia)	n.d.	n.d.	20–50 pmol/gFW	Morrison et al., 2017
Plants	Soybean (*Glycine max*) (seed; R6)	2MeSZ, 2MeSZR	1–15 ng/gFW	5–30 ng/gFW	Kambhampati et al., 2017
	*Arabidopsis thaliana* Col (seed)	2MeSZ, 2MeSIP, 2MeSZR	100–150 pmol/gFW	10,000–15,000 pmol/gFW	Butler, 2019
Animals	*Rhinusa pilosa* (adult)	2MeSZ, 2MeSZR	1,500–2,000 pmol/gFW	2,000–2,500 pmol/gFW	Andreas et al., 2020
	Dog (*Canis familiaris*) (stomach)	2MeSiP, 2MeSZR, 2MeSiPR	15–20 pmol/gFW	50–100 pmol/gFW	Seegobin et al., 2018
	HeLa cells (*Homo sapiens*)	2MeSZ, 2MeSiP, 2MeSZR, 2MeSiPR	1,000–5,000 pmol/mL	5,000–10,000 pmol/mL	Aoki et al., 2019b

*Using HPLC-MS/MS analysis at the Water Quality Centre, Trent University, example organisms of the Prokaryotae (bacteria) and the Eukaryotae (protists, fungi, plants, and animals) were scanned for presence of 2MeSZ, 2MeSZR, 2MeSiP, and 2MeSiPR. 2MeS-CKs were detected in the representatives of all the analyzed kingdoms except for fungi. n.d., not detected.*

2-Methylthio-Zeatin riboside (2MeSZR) and 2-methylthio-Zeatin (2MeSZ) accumulated in the infected cobs during the later stages of maize infection by *U. maydis* but were not present in aseptic fungal cultures or in control plant tissue. This finding suggests there is a fungal-stimulated, plant-origin of 2MeS-CKs that may be involved in promoting tissue proliferation around the site of infection (Morrison et al., 2015a).

**FIGURE 3:**
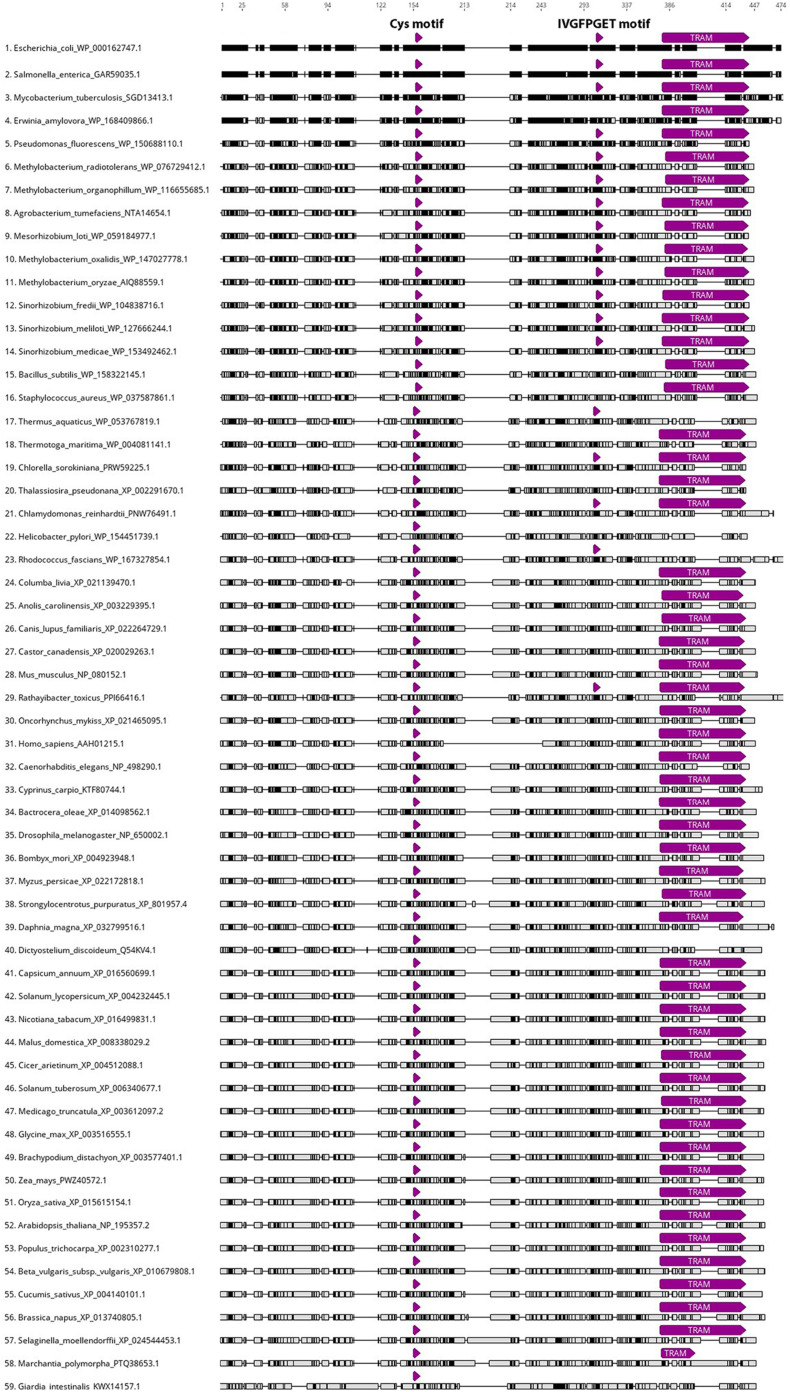
MAFFT alignment of 59 miaB-like protein sequences including representatives of *Archaea*, *Bacteria*, *Protista*, *Planta*, and *Animalia* kingdoms. All amino acid sequences were trimmed to the length of *E. coli* miaB (WP_000162747.1; 1–474 aa). WP_000162747.1 accession was set as reference sequence and all aligned sequences were sorted by the number of differences to reference sequence. Black color indicates regions identical to those of *E. coli* miaB, gray color indicates differences in the aligned sequences from miaB protein. The locations of three highly conserved miaB regions are highlighted by the purple arrows: Cys motif (CxxxCxxC) – involved in methylthiolation of the prenylated adenine in tRNA 37 position, was identified in all the analyzed sequences; IVGFPGET motif was found in 18 accessions, including representatives of *Archaea*, *Bacteria* and *Protista*; TRAM domain, responsible for nucleic acid binding, was present in 54 out of the 59 aligned sequences. The alignment, motif search and visualization were performed using Align/Assemble function in Geneious Prime 2020.2.3.

Apart from fungi, *miaB* homolog genes can be found across all kingdoms of life, indicating their evolutionarily conserved character and universal roles in posttranscriptional and posttranslational RNA modifications (Anantharaman et al., 2002). Figure 3 presents a MAFFT alignment (Geneious Prime 2020.2.3) of the characterized and predicted miaB-like proteins in model and non-model species from *Archaea*, *Bacteria*, *Protista*, *Planta*, and *Animalia*. The top candidates were selected based on the highest sequence similarity with *E. coli* miaB protein sequence (accession no. WP_000162747.1) using non-redundant BLASTp searches on 59 selected taxids. Searches were done using the BLOSUM62 matrix and default parameters.^1^ A signature miaB motif that has three cysteine residues spaced by 3 and 2 amino acids (CxxxCxxC; Figure 3), and that is responsible for iron binding in the process of thiolation (Esberg et al., 1999), was present in all the analyzed sequences. Another highly conserved miaB motif (IVGFPGET; Figure 3; Esberg et al., 1999) was found in 18 species including representatives of Archaea, Bacteria and Protists. The presence of the TRAM domain (PF01938) responsible for nucleic acid binding, has been identified in 54 of the 59 aligned sequences^2^; however, the remaining five accessions revealed similar sequence structure to those of TRAM domains from the other analyzed taxons (Figure 3).

The *A. thaliana* homolog of bacterial *miaB* gene is known as AT4G36390. Structurally, AT4G36390 resembles *miaB* as it contains a C terminal RNA-binding TRAM domain and functions similarly to bacterial tRNA methylthiotransferases (Dabravolski, 2020). AT4G36390 is predicted to localize mainly to the mitochondria and carry an [Fe-S]; however, much of its role remains unknown (Dabravolski, 2020).

In mammals, cyclin dependent kinase 5 regulatory subunit-associated protein 1 (CDK5RAP1) is a type of radical SAM enzyme that reductively cleaves S-adenosyl-L-methionine (Atta et al., 2010), with homology to *miaB* (Kaminska et al., 2008; Reiter et al., 2012). CDK5RAP1 is responsible for methylthiolation of tRNA-bound CKs and has roles in altering stability of tRNA molecules, interactions with ribosomes and translation (Jenner et al., 2010; Horvath and Chinnery, 2015). The presence of unbound 2MeS-CK metabolites was recently reported from the surveyed canine tissues and human cell cultures (Seegobin et al., 2018; Aoki et al., 2019b).

### Hydroxylation of the Isopentenyl Chain of tRNA-Bound 2MeSiP-CKs: tRNA-ms2io6A37-Hydroxylase: MiaE/CYP450

A further step of the 2MeS-CK production via tRNA degradation pathway involves hydroxylation of the isopentenyl side chain which can occur before or after methylthiolation (Dabravolski, 2020) (Figure 2). The biosynthesis of tRNA-bound ms^2^io^6^A (2MeS*c*ZR) in *S. typhimurium* involves multiple enzymatic activities and their corresponding genetic loci of which *miaE* is responsible for the hydroxylation step (Persson and Björk, 1993). The tRNA-(ms^2^io^6^A37)-hydroxylase is present in *Salmonella* under anaerobic conditions; but the hydroxylation reaction does not occur in the absence of oxygen, indicating that it depends on the presence of molecular oxygen (Buck and Ames, 1984).

Bacterial MiaE hydroxylates a terminal methyl group of ms^2^i^6^A (2MeSiPR) to form ms^2^io^6^A (2MeS*c*ZR). Cloning the *miaE* gene that encodes the tRNA hydroxylase from *S. typhimurium* and complementing the *E. coli* strain that is naturally deficient in ms^2^io^6^A hydroxylation gene increased the rate of ms^2^i^6^A37 (2MeSiPR) hydroxylation by approximately 250-fold (Persson and Björk, 1993). It has been suggested that the *miaE* gene may encode either the tRNA-(ms^2^io^6^A37)-hydroxylase or a cofactor necessary for the hydroxylation reaction. However, it is unlikely that *miaE* encodes a cofactor needed for only one reaction, and even less likely that *E. coli* would have kept the hydroxylating enzyme through evolution despite the absence of the necessary cofactor; therefore, it seems more probable that the *miaE* gene encodes the tRNA-(ms^2^io^6^A37)-hydroxylase itself (Persson and Björk, 1993).

Fungi lack the *miaE* gene as it is not found in eukaryotes (Persson and Björk, 1993; Frébort et al., 2011). In plants, conversion of iPR and 2MeSiPR to *c*Z and 2MeS*c*ZR, respectively, occurs through hydroxylases, which function similarly to bacterial MiaE enzymes (Takei et al., 2004). BLASTp searches could not identify corresponding hydroxylase homologs in mammalian candidates (Seegobin et al., 2018). However, CYP450 enzymes that are involved in hormone metabolism in mammals (Nebert and Russell, 2002; Schmidt et al., 2008) remain to be investigated for their potential role as *cis*-hydroxylating agents, especially regarding the previously detected 2MeSZR metabolites in both canine tissues and human cells (Seegobin et al., 2018; Aoki et al., 2019b).

## 2MeS-CK Metabolites Associate With the Profiles of More Commonly Studied CKs Across the Kingdoms of Life

Unbound 2MeS-CKs are present in biological systems as a result of degradation of the tRNA molecules modified by the set of genes described in the previous sections. The genes responsible for subsequent steps of 2MeS-CK biosynthesis and the effects of tRNA modifications leading to the formation of bound 2MeS-CKs in microbes and mammals have been known for decades; but the highly sensitive methods of HPLC-MS/MS that enable the precise detection and quantification of unbound, hydrophobic 2MeS-CK metabolites from biological samples have only been available for the last 10 years (Tarkowski et al., 2010; Šimura et al., 2018; Kisiala et al., 2019). Table 1 is a summary of the previously published HPLC-MS/MS results which confirm a recurring presence of 2MeS-CKs among species from various kingdoms of life including microbes, plants, and animals.

To date, most research on CK molecules have investigated plant classical CKs (unmodified iP-, *t*Z-, *c*Z-, and DZ-types) while not much interest has been put into explaining the presence of unbound 2MeS-CKs in biological systems (Großkinsky et al., 2016). This, in consequence, resulted in a general lack of studies involving mutants impaired in the function of 2MeS-CK biosynthetic genes, as compared to *IPT*, *LOG*, or *CKX* modifications, creating additional challenges for understanding the methylthiolated CK compounds. Nevertheless, the available CK profiling data on *miaB* knock-out mutant of *E. coli* (JW0658-1) revealed a lack of 2MeS-CKs while, simultaneously, the levels of iPRP were considerably increased in the culture lysate (Daudu et al., 2019). These findings support other evidence from genetic studies that the pool of available prenyl-tRNA – the precursor molecule of the classical CKs originated via tRNA degradation pathway – is also the source of unbound 2MeS-CKs (Esberg et al., 1999; Morrison et al., 2017; Figure 2). The metabolite profiles reported for microbial, plant and animal-derived 2MeS-CKs often reflect the close association between this group of compounds and select, classical CK types (particularly, iP and its precursor forms), which implies that they originate from a common precursor in the tRNA degradation pathway.

*Methylobacterium* strains are plant-associated bacteria well known for their plant growth promoting characteristics and unique capabilities to synthesize high levels of biologically active *t*Z-type CKs. The study of CK profiles of eight *Methylobacterium* isolates revealed that strains secreting the highest levels of 2MeS-CKs (specifically 2MeSZ) simultaneously had low *t*Z production (i.e., *M. organophillum* NBRC103119: *t*Z – 12.4 pmol/10 mL, 2MeSZ – 119.6 pmol/10 mL, and *M. oryzae* LMG23582(T): *t*Z – 212.9 pmol/10 mL, 2MeSZ – 39.9 pmol/10 mL) (Jorge et al., 2019).

In plants, the analysis of CK profiles during the reproductive development of 27 field grown soybean cultivars revealed an association between the levels of 2MeS-CKs and CK nucleotides. Namely, the cultivars characterized by the higher 2MeSZ levels in their pods at R4 growth stage contained lower concentrations of iPRP while high iPRP production was associated with the lower 2MeSZ levels in the developing pods (Pearson’s *r* = −0.467) (Kambhampati et al., 2017). A clear, offsetting balance between the levels of CK nucleotides and 2MeSCKs occurs during the reproductive growth stages of *A. thaliana* WT as well as its two tRNA-IPT mutants, *ipt2* and *ipt9* (Butler, 2019). In detail, in immature siliques, *Arabidopsis* plants contained prominent levels of iPRP and a relatively low concentrations of a single 2MeS-CK form (2MeSZ). On the other hand, CK profiles of maturing seeds lacked any nucleotide CK precursors while considerable increases in the concentrations of 2MeS-CK derivatives [2MeSZ, 2MeSZR and 2-methylthio-isopentenyladenine (2MeSiP)] were observed (Butler, 2019).

These types of inverse associations were also characteristic for the HPLC-MS/MS profiles of CKs reported from representatives of six orders of Insecta (Andreas et al., 2020). For example, larvae of two stem-boring beetle species, *Mecinus janthinus* and *Mecinus janthiniformis*, demonstrated highly elevated concentrations of nucleotide precursors of the classical CKs and only trace levels of 2MeS-CK derivatives. The adult forms of these two weevils had unusually high levels of 2MeS-CKs (2MeSZ and 2MeSiP), and minimal concentrations of the classical CKs (*t*Z- and iP-type) suggesting there were different activities of the genes in the tRNA modification pathway during subsequent stages of insect development. Similar associations were observed among other insect forms and species (Andreas et al., 2020).

A negative association between the levels of 2MeS-CKs and classical CKs was reported from analyses of mammalian samples (Seegobin et al., 2018). The presence of 2MeS-CKs was identified in 8 out of 21 tested canine organs and tissues, which included: the inner and outer kidney medulla, stomach, appendix, small and large intestine, bile and thyroid. In most of the analyzed organs, iP-type CKs were the predominant CK forms while 2MeS-CKs were generally found at much lower levels. However, in bile, considerably higher concentration of 2MeS-CKs were detected, which was accompanied by lower levels of iP-type CKs (i.e., thyroid: 2MeS-type CKs – 3.82 pmol/gFW, iP-type CKs – 1392.75 pmol/gFW; bile: 2MeS-type CKs – 229.96 pmol/gFW, iP-type CKs – 114.68 pmol/gFW) (Seegobin et al., 2018).

## Evidence of the Potential Physiological Roles of 2MeS-CKs

Despite the availability of mass spectrometry methods for the analysis of 2MeS-CKs (Tarkowski et al., 2010; Kisiala et al., 2019) and emerging number of reports of their presence in different biological systems (including mammals, plants, pathogen-host associations, bacteria, and algae), the functional significance of 2MeS-CKs continues to remain poorly understood (Esberg et al., 1999; Morrison et al., 2015a; Kambhampati et al., 2017; Daudu et al., 2019; Koshla et al., 2019). Most knowledge about the role of 2MeS-CKs stems from their occurrence in post translational tRNA modifications which have a role in translational efficiency, fidelity, and specificity of codon/anticodon interactions (Persson and Björk, 1993; Golovko et al., 2000; Koshla et al., 2020). Deletion of key genes in the methylthiolation pathway can result in aberrant phenotypes most often related to impacts on translation (Koshla et al., 2019). However, the impact of the released, methylthiolated CK compounds on the physiology of organisms has yet to be fully understood.

Koshla et al. (2019, 2020) examined the impact of *miaA, B* and *AB* null mutants in *Streptomyces albus* showing that *miaA* and *B* deficiency caused significant changes in morphology of *Streptomyces* and its secondary metabolome profile. Likely caused by less efficient translation, the *miaAB* mutant was devoid of endogenous antibacterial activity while the parent *miaA* mutant still displayed some antibacterial activity (Koshla et al., 2019). *miaB* mutants had delayed biomass production and *miaAB* mutants produced sparse aerial hyphae without spore chains compared to the parental mutant *miaA* (Koshla et al., 2020), suggesting that, even in the absence of miaA, miaB functions to maintain cell cycle and morphology. Phenotypic discrimination between the mutated bacterial lines was highly conditional and only possible on specific media during early stages of their lifecycle; indicating that the impact of methylthiolation may be specific to certain stages of the growth cycle under defined nutrient conditions (Koshla et al., 2020). Although the levels of unbound 2MeS-CKs were not analyzed, this study suggests that the removal of genes responsible for thiolation impacts total free 2MeS-CKs and thereby also has an impact on the overall development of the bacteria (Koshla et al., 2020).

Similar to bacteria, knockdown mutations of enzymes responsible for the methylthiolation of tRNA in mammals resulted in changes to cell cycle. Mammalian CDK5RAP1 is related to the regulation and progression of the M phase of the cell cycle (Wang et al., 2015). Deficiency of the CDK5RAP1 enzyme (a homolog of bacterial miaB) in the knockdown mutation of human breast cancer cell line MCF-7 suppressed tumor growth through cell arrest at the G2/M phase and induced cell apoptosis (Wang et al., 2015). Cytokinins have antitumor effects, with exogenous iPR treatment causing autophagic cell death; however, CDK5RAP1 reduced the antitumor effect of endogenous iPR by converting it to 2MeSiPR and protecting glioma initiating cells (GICs) from autophagy triggered by iPR toxicity. Isopentenyladenosine was not converted directly to 2MeSiPR when it was exogenously applied to GICs (Yamamoto et al., 2019).

The hypermodifications of tRNA not only aid in translation and cell cycle functioning, but after degradation of the modified tRNAs, they act as small molecules able to trigger physiological effects and activate CK receptors (Daudu et al., 2017, 2019). The role of histidine kinase (HK) CK receptors from *Malus domestica* (apple tree) in the perception of classical and methylthiolated CKs were examined utilizing a histidine kinase deletion mutant in yeast (*sln1* deletion) (Daudu et al., 2017). Complementation assays and examining yeast cell growth in response to CK addition revealed that MdCHK2 have higher sensitivity to iP and 2MeSiP (1 nM) relative to other CKs tested. Other examined MdCHKs also responded to 2MeS-CKs (5 nM–5 μM depending on receptor) indicating that 2MeS-CKs can be perceived by CK receptors and trigger growth responses (Daudu et al., 2017).

The yeast mutant strain *sln1*Δ-MdCHK was further utilized as a CK biosensor and was exposed to extracts from a variety of organisms to detect their production of CKs (Daudu et al., 2019). When exposed to supernatant extracts from *E. coli miaB* deletion mutant, yeast growth was triggered but was considered to be less than the wildtype. Yeast growth was not decreased due to changes in total level of CKs as these were similar between the wildtype (16.6 pmol/mL) and *miaB* mutant (17.9 pmol/mL). The *E. coli miaB* deletion strain had higher levels of iPRP and iP relative to the WT strain while it was suggested that the limited yeast growth was due to reduction in 2MeSiP which was not detected in the *miaB* deletion mutant (Daudu et al., 2019).

The ability of receptors to respond to 2MeS-CKs suggests these unique CKs can trigger downstream impacts such as growth changes. Correlations between 2MeS-CK levels and observed phenotypes can indicate a potential role or influence that 2MeS-CKs may have on a system. In a field trial sampling of twenty-seven soybean cultivars high quantities of 2MeSZR and 2MeSZ were detected at all three stages of seed filling (R4,5,6) of high yielding varieties. There was also a positive correlation of 2MeSZ with all yield components measured suggesting 2MeS-CKs may play a role in seed filling and subsequent yield formation in soybean (Kambhampati et al., 2017).

In plant-microbe interaction systems, 2MeS-type CKs are often suggested to be of microbial origin. Cytokinins, including bacterially produced 2MeS-CKs play a role in the dormancy of annual ryegrass (*Lolium rigidum*) seeds (Goggin et al., 2015). Removal of the bacterial symbiont resulted in seeds being unable to break dormancy in the dark, suggesting that a complex integration between plant hormones and those produced by bacterial microflora, including 2MeS-CKs, play an important role in controlling seed maturation in *L. rigidum* (Goggin et al., 2015).

Spikes in 2MeS-CKs have been detected during host-pathogen relations where increased plant growth is a disease symptom. *A. thaliana* infection by a bacterial phytopathogenic strain of *R. fascians* had the same spectrum of CKs as the non-pathogenic counterpart; yet the former had higher levels of 2MeS*c*Z, iP and *c*Z (Pertry et al., 2009). The authors suggested that 2MeS-CKs may be less cytotoxic than classical CKs which could account for their accumulation during infection (Pertry et al., 2009). The low but consistent presence of 2MeS-CKs may be important in eliciting plant responses during disease development (Giron and Glevarec, 2014). The accumulation of *c*Z and 2MeS-CKs in infected plant tissues and the transient peak in the levels of iP, 2MeSiP, and 2-methylthio-*trans*Zeatin (2MeS*t*Z) in the early interaction phase hinted at the biological significance of the identified bacterial 2MeS-CKs (Pertry et al., 2009). These characteristics also suggest that 2MeS-CKs may be more resistant to CK degrading enzymes (CKX) and are capable of escaping attempts by the host to balance CKs (Morrison et al., 2015a). Therefore, 2MeS-CKs are thought to help hijack the plant machinery while avoiding deleterious effects on plant development and activation of CK mediated plant defenses (Giron and Glevarec, 2014). Overall, 2MeS-CKs may be responsible for continuous tissue proliferation and symptom maintenance during plant-pathogen interaction (Pertry et al., 2009). Observations of the *Zea mays – U. maydis* plant-pathogen system led to the similar conclusion as previously suggested for the 2MeS-CK effect in *Arabidopsis – R. fascians* interaction. Although the fungus *U. maydis* does not produce 2MeS-CKs separate from the plant, during the *Zea mays – U. maydis* relation an accumulation of 2MeS-CKs (specifically 2MeSZR and 2MeSZ) occurred in the infected plant tissue during the later stages of cob infection (days 20-28) while these CKs were not detected in the control cobs (Morrison et al., 2015a, 2017).

The contribution of CK interplay was examined in the human pathogen, *Mycobacterium tuberculosis*, the causative agent of tuberculosis. This bacterium secretes several iP- and *t*Z-type CKs including 2MeSiP, 2MeSiPR and 2MeS*t*ZR (Samanovic et al., 2015). A higher activity of *M. tuberculosis* LOG-like enzyme, Rv105, results in CK accumulation including 2MeSiP. That, in turn, increases sensitivity of bacterial strains to nitric oxide, a defense response from the host cells, detrimental to pathogen survival. These results suggest that among other CKs, 2MeS-CKs play a role in sensitizing the pathogen to the activation of host defense mechanisms (Samanovic et al., 2015).

While most studies focus on the impact of gene deletion and the resulting effects which might then be rescued through gene complementation (Koshla et al., 2020), few have added exogenous 2MeS-CKs to rescue deletion phenotypes or applied exogenous methylthiol CK derivatives to examine their impact on a biological system.

The HPLC-MS/MS analysis of CK profiles in microalgae *Chlorella vulgaris* revealed that 2MeS-CKs were the prevalent CKs in both culture supernatant and cell pellet, with 2MeSZ being the most abundant in the pellet (4.7 pmol/g) and at high concentrations in the supernatant (220.7 pmol/g) followed by other methylthiol conjugates (Ramphal, 2016). To understand the role of 2MeS-CKs in *C. vulgaris* cell cycle, the effect of exogenous 2MeSZ treatment on the cell growth and select metabolite production were examined. Culture supplementation with 2MeSZ resulted in growth stimulation and had an impact on the microalgae fatty acid profiles in a manner comparable to those of the other active CKs that were tested (*t*Z, benzyladenine). In particular, significant increases in linolenic acid (18-carbon chain with three double bonds; 18:3) occurred at 10^–7^ and 10^–6^ M 2MeSZ concentrations; 204 and 457% increase relative to the control, respectively (Ramphal, 2016). It indicates that the addition of exogenous methylthiol CKs can impact growth as well as fatty acid metabolism in algae. Modified secondary metabolite production was also a result of the deletion of *miaB* in the bacterium, *S. albus* (Koshla et al., 2020). These studies suggest that the methylthiolated CK compounds are not only necessary for efficient translation but likely are also involved in activating downstream metabolic pathways.

## Conclusion

Physiological roles of classical CKs (unmodified iP, *t*Z, *c*Z, and DZ and their derivatives) have been studied extensively to help uncover the functions of these signaling molecules in different aspects of plant physiology. In parallel, extensive knowledge exists for genes involved in tRNA modifications that lead to the production of tRNA-bound 2MeS-CKs, especially in bacterial and mammalian systems. However, not much is known about the fate and possible function of the unbound 2MeS-CKs, derived via degradation of the modified tRNA molecules in biological systems.

There is a compelling need to further study this most elusive group of CKs as evidenced by their frequent detection, which is often at high concentrations when found in different plant organs and at different developmental stages; moreover, there is an increasing number of reports on 2MeS-CK presence in other, non-plant organisms. Suggestions of the potential metabolic activities of unbound 2MeS-CKs have been made previously. The limited physiological data available indicate that 2MeS-CKs may play a role that is similar to classical CKs; whereby, 2MeS-CKs impact cell division as they are often detected in systems where active proliferation occurs, like plant-pathogen disease development and mammalian cancer cells. In addition, 2MeS-CKs may be able to reduce the pool of more active iP-type CKs resulting in production of less cytotoxic forms of CKs. As discussed above, conversion of iPR in mammals to 2MeSiPR reduced the anticancer effect of iPR on cells thus decreasing the potency of the CK application. Likewise, production of 2MeS-CKs during plant-pathogen disease development may allow for persistent accumulation of forms that are more resistant to degradation and, therefore, can act over a longer period of time. Finally, exogenous application of 2MeS-CKs can impact cell metabolism and growth as seen in *C. vulgaris*.

The potential impact of 2MeS-CKs should no longer be overlooked. Their functional role needs to be better understood and further investigation into the deletion of key genes in the methylthiolation pathways as well as the exogenous applications of 2MeS-CKs should be continued to determine the physiological roles of these compounds. However, parsing out the translation-efficiency effects of bound 2MeS-CKs and physiological effects of unbound 2MeS-CKs may prove to be quite difficult. With this in mind, the development of CK mutants impaired in functioning of 2MeS-CK biosynthesis genes might create significant challenges and the possible, strong pleiotropic effects of the mutations targeting the genes involved in tRNA modifications have to be considered. tRNA-bound 2MeS-CKs play critical roles in diverse aspects of RNA metabolism and the effect of their disturbance might be difficult to separate from the potential physiological roles of unbound 2MeS-CKs obtained via tRNA degradation. New approaches that separate effects of tRNA-bound and unbound-2MeS-CKs will have to be developed to overcome that challenge, of fully understanding the often-prevalent and enigmatic 2MeS-CKs.

## Author Contributions

MG and AK wrote the first draft of the manuscript. EM and RE wrote sections of the manuscript. All authors contributed to manuscript revision, read and approved the submitted version.

## Conflict of Interest

The authors declare that the research was conducted in the absence of any commercial or financial relationships that could be construed as a potential conflict of interest.

## Footnotes

1 https://www.ncbi.nlm.nih.gov/protein/accessed August 2020

2 https://pfam.xfam.org/and NCBI Batch CD-Search Tool accessed August 2020
